# *Egr2::Cre* Mediated Conditional Ablation of Dicer Disrupts Histogenesis of Mammalian Central Auditory Nuclei

**DOI:** 10.1371/journal.pone.0049503

**Published:** 2012-11-12

**Authors:** Elena Rosengauer, Heiner Hartwich, Anna Maria Hartmann, Anya Rudnicki, Somisetty Venkata Satheesh, Karen B. Avraham, Hans Gerd Nothwang

**Affiliations:** 1 Department of Neurogenetics, Carl von Ossietzky University Oldenburg, Oldenburg, Germany; 2 Department of Human Molecular Genetics and Biochemistry, Sackler Faculty of Medicine, Tel Aviv University, Tel Aviv, Israel; 3 Center for Neuroscience, Carl von Ossietzky University Oldenburg, Oldenburg, Germany; University of Washington, United States of America

## Abstract

Histogenesis of the auditory system requires extensive molecular orchestration. Recently, *Dicer1*, an essential gene for generation of microRNAs, and miR-96 were shown to be important for development of the peripheral auditory system. Here, we investigated their role for the formation of the auditory brainstem. *Egr2::Cre*-mediated early embryonic ablation of *Dicer1* caused severe disruption of auditory brainstem structures. In adult animals, the volume of the cochlear nucleus complex (CNC) was reduced by 73.5%. This decrease is in part attributed to the lack of the microneuronal shell. In contrast, fusiform cells, which similar to the granular cells of the microneural shell are derived from *Egr2* positive cells, were still present. The volume reduction of the CNC was already present at birth (67.2% decrease). The superior olivary complex was also drastically affected in these mice. Nissl staining as well as Vglut1 and Calbindin 1 immunolabeling revealed that principal SOC nuclei such as the medial nucleus of the trapezoid body and the lateral superior olive were absent. Only choline acetyltransferase positive neurons of the olivocochlear bundle were observed as a densely packed cell group in the ventrolateral area of the SOC. Mid-embryonic ablation of *Dicer1* in the ventral cochlear nucleus by *Atoh7::Cre*-mediated recombination resulted in normal formation of the cochlear nucleus complex, indicating an early embryonic requirement of *Dicer1*. Quantitative RT-PCR analysis of miR-96 demonstrated low expression in the embryonic brainstem and up-regulation thereafter, suggesting that other microRNAs are required for proper histogenesis of the auditory brainstem. Together our data identify a critical role of Dicer activity during embryonic development of the auditory brainstem.

## Introduction

Normal hearing requires proper development of the central auditory system. After transduction of acoustic signals in the cochlea, all auditory information is transmitted by spiral ganglion neurons to the central auditory system for signal processing and perception [1], [2]. The first central structure to process auditory information is the cochlear nucleus complex (CNC) [1], [2]. It is the sole intermediary between the periphery and higher centers of the central auditory system and distributes information to different ascending pathways [1]. Anatomically, the CNC is a tripartite structure consisting of the anteroventral cochlear nucleus (AVCN), the posteroventral cochlear nucleus (PVCN), and the dorsal cochlear nucleus (DCN) [3]. DCN neurons primarily project to the inferior colliculus [3]. The major targets of the VCN are the lateral superior olive (LSO), the medial superior olive (MSO), and the medial nucleus of the trapezoid body (MNTB), which constitute the major nuclei of the SOC [3], [4]. The SOC represents the first binaural processing center and participates in sound localization by computing interaural time and level differences [3], [5]. In addition, neurons within the SOC give rise to the olivocochlear (OC) bundle, an efferent feedback system, that modulates cochlear function [6].

Due to their prominent role in auditory information processing, both the CNC and SOC have been extensively characterized with respect to electrophysiological properties [7]–[9], molecular profiles [10]–[13], and maturation processes [14], [15]. Much less is known concerning their histogenesis. Genetic analyses in mice identified rhombomeres (r) 2 to 5 as the major source of the CNC [16], [17], and established r3 and r5 as the origin of many principal SOC neurons [18]. However, the genetic program, underlying histogenesis of these two auditory structures, is largely unknown. Only recently, the proneural basic helix-loop-helix transcription factor Atoh1 was shown to be important for histogenesis of the CNC and SOC [18].

Several studies identified a central role of microRNAs (miRNA) for proper formation and function of neuronal circuits. miRNAs are small non-coding RNAs that regulate gene expression on the transcriptional and posttranscriptional level [19]–[21]. They are generated *in vivo* from long primary (pri-) miRNAs, which require final processing by Dicer, a ribonuclease type III endonuclease which recognizes double-stranded RNA molecules [22]. Dicer activity results in mature miRNAs of 21–27 nts that interact with complementary mRNA sequences. Target recognition is based on the complementarity between the seed region (nucleotides 2–8) of a miRNA and the mRNA [23]. Binding to the target mRNA results in translational repression or mRNA cleavage, thereby offering a novel layer of gene regulation [21], [24], [25].

The number of Dicer-like proteins varies among organisms. Whereas organisms such as *Drosophila melanogaster* [26], plants [27], and fungi [28] possess multiple Dicer-like proteins, mammals have only one gene, *Dicer1* [29]. Ablation of *Dicer1* in mice severely disrupts miRNA pathways, resulting in loss of the inner cell mass of the blastocyst and embryonic arrest at E7.5 [29]. To further study the role of miRNAs and Dicer, conditional alleles of *Dicer1* have been generated [30]–[32]. This approach identified numerous functions of miRNAs in the nervous system such as their regulatory role in neurogenesis, synaptogenesis, differentiation, and plasticity [21], [24], [33], [34]. These small RNAs were also shown to regulate many sensory systems such as the visual [35]–[37] and olfactory systems [38], [39], taste [40], [41], CO_2_ sensing [42], and pain perception [43]. Studies in the auditory system revealed an essential role of miRNAs in the cochlea. Early embryonic (∼E8.5) ablation of *Dicer1* in the otic placode using a *Foxg1::Cre* driver line resulted in near complete loss of the ear and the neurosensory epithelium [44], and depletion using a *Pax2::Cre* driver line gave rise to disorganized inner and outer hair cell rows and lack of innervation of the sensory epithelium by the auditory nerve [45]. Later ablation of *Dicer1* at E14.5 resulted in postnatal malformation of hair cells such as loss or disorganization of stereocilia [46]. Furthermore, mutations in miR-96 are associated with peripheral hearing loss both in man and mouse [47]–[49]. In the mouse this is caused by arresting physiological and morphological development of cochlear hair cells around birth [47]–[49].

Here we investigated the role of *Dicer1* for the development of the auditory brainstem by analyzing two different mouse lines with differentially timed disturbance in miRNA function. In one mouse line, *Egr2::Cre;Dicer1^fl/fl^*, Dicer is deleted in r3 and r5 from early embryonic stages on, whereas in the other mouse line, *Atoh7: Cre;Dicer1^fl/fl^*, the enzyme is deleted from mid embryonic stages on in bushy cells of the VCN. Anatomical and immunohistochemical analyses of these mouse lines identified a critical early role of Dicer in histogenesis of the CNC and SOC. Expression analyses of miR-96 suggested the involvement of other miRNAs in histogenesis of the auditory brainstem.

## Materials and Methods

### Ethics Statement

All protocols were in accordance with the German Animal Protection law and approved by the local animal care and use committee (LAVES, Oldenburg 33.9-42502-04-10/0235) and the Animal Care and Use Committee of Tel Aviv University (M-09-057). Protocols also followed the NIH Guide for the Care and Use of Laboratory Animals. All efforts were made to minimize suffering.

### Animals

The *Dicer1^fl/fl^* mouse [31], the *ROSA26R* mouse [50], and the Cre-driver lines *Egr2::Cre* [51] and *Atoh7::Cre* [52] have been described previously. Littermates that carry the incomplete combination of alleles served as wild type controls. All animals used for these experiments were maintained on mixed backgrounds.

### Immunodetection, β-galactosidase Staining, and Histology

Rabbit anti-Vglut1 antibody was a generous gift from Dr. S. El Mestikawy (Creteil, Cedex, France) [53], rabbit anti-ChAT was obtained from Millipore (Schwalbach, Germany), and goat anti-Calbindin 1 from Swant (Marly, Switzerland). Antibodies were diluted 1∶1,000 (anti-Vglut1), 1∶150 (anti-ChAT), and 1∶5,000 (anti-Calbindin 1) with carrier solution containing 1–2% bovine serum albumin, 10% goat serum (for anti-Vglut1), and 0.3–0.5% Triton X-100 in phosphate-buffered saline (PBS) (150 mM NaCl, 10 mM Na-phosphate, pH 7.4). Sections were incubated overnight with agitation at 7°C. They were then rinsed three times for 10 min with washing solution (0.5% Triton X-100 and 0.1% saponin in PBS, again transferred in carrier solution and treated with the secondary antibody (diluted 1∶500 to 1∶1,000, Invitrogen). After several washes in washing solution and PBS, images were taken with a BZ 8100 E fluorescence microscope (Keyence, Neu-Isenburg, Germany).

For β-galactosidase staining, *Egr2::Cre*;*ROSA26R* mice were perfused by 4% paraformaldehyde. Brains were then cryosectioned at 60 µm in free floating conditions and stained in X-gal solution (3 mg/ml X-gal, 7.2 mM Na_2_HPO_4_, 2.8 mM NaH_2_PO_4_, 150 mM NaCl, 1 mM MgCl_2_, 3 mM K_3_(Fe(CN)_6_), 3 mM K_4_(Fe(CN)_6_, 1% NP-40) for 24–48 hours at 37°C.

Nissl staining was performed on 30-µm-thick sections. The volume of the auditory nuclei was calculated by multiplying the outlined area with the thickness of each section. Two animals were used for each genotype at each age, resulting in 4 auditory nuclei per analysis. Statistical analysis was performed using the non-parametric Mann-Whitney U test.

### Tissue Preparation and RT-PCR Analysis

Mice were anesthetized with 7% chloral hydrate (60 µl/g body weight) and decapitated. The brainstem was dissected and 250-µm-thick coronal slices, containing the CNC or SOC, were cut with a vibratome (Leica VT 100 S, Leica, Nussloch, Germany) under binocular control. Collected tissue was stored in RNAlater (Ambion, Darmstadt, Germany) at -20°C. After DNA extraction from the tissue, the following primer pairs were used for genotyping: 460R GTACGTCTACAATTGTCTATG, 23F ATTGTTACCAGCGCTTAGAATTCC and 458F TCGGAATAGGAACTTCGTTTAAAC [31]. Total RNA extraction from tissue was performed using acid guanidinium thiocyanate-phenol-chloroform extraction [54]. After reverse transcription, PCR reactions were performed with the primer pair: exon20fn GACACTGTCAAATGCCAGTG and exon24rev GCCTTGGGGACTTCGATATC. This primer combination amplifies a 1,500 bp long fragment from wild type *Dicer1* mRNA and a 360 bp fragment of the truncated mRNA after genetic recombination. PCR products were separated by standard agarose gel electrophoresis and GelRED (Genaxxon, Ulm, Germany) to stain nucleic acids.

For miR-96 expression analysis, the brainstem was dissected from C57BL/6 mice at E18, P0 and P25 and small RNA was extracted using the miRNeasy Mini Kit (QIAGEN). cDNA was transcribed using the High Capacity cDNA Reverse Transcription Kit (Applied Biosystems) and miR-96 taq-man primer UUUGGCACUAGCACAUUUUUGCU designed by Applied Biosystems. For the qRT-PCR reaction, miR-96 probe (Applied Biosystems) and FastStart Universal Probe Master Mix (Roche) were used in the StepOne Plus qRT-PCR machine (Applied Biosystems). U6B served as endogenous control. At least 3 experiments were conducted, with triplicates.

## Results

### *Egr2::Cre-*mediated Loss of *Dicer1* Disrupts Formation of the CNC

**Figure 1:**
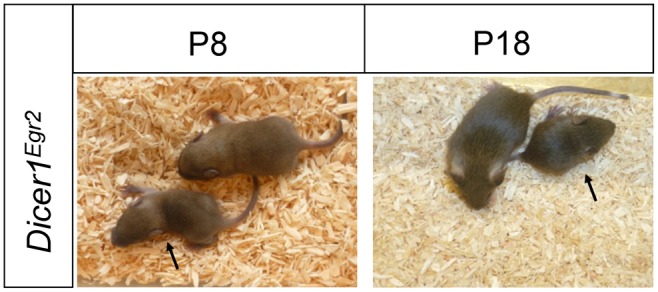
Smaller size of *Dicer1^Egr2^* mice. Compared to wild type littermates, *Dicer1^Egr2^* mice (black arrows) were smaller and the difference in size increased with age. P, postnatal day.

To determine the role of miRNAs for proper development of the auditory brainstem, we used a *Dicer1^fl/fl^* mouse line with *loxP* sites flanking exons 23 and 24 of the *Dicer1* gene [31]. These two exons encode the majority of both RNase III domains. The mouse line was paired with the Cre-driver line *Egr2::Cre* (aka *Krox20::Cre*), which drives Cre expression specifically in r3 and r5 [51]. These two rhombomeres are the major source of CNC and SOC neurons [16], [18]. *Egr2::Cre;Dicer1^fl/fl^* animals *(Dicer1^Egr2^* in the following) were born in a non-mendelian ratio and became smaller than their littermates with increased age (Fig. 1). In addition, few *Dicer1^Egr2^* animals survived beyond the first postnatal days. One explanation might be malfunction of the respiratory system, as Egr2 positive cells contribute to the parafacial respiratory group [55], [56], which is part of the brainstem respiratory neuronal network.

**Figure 2:**
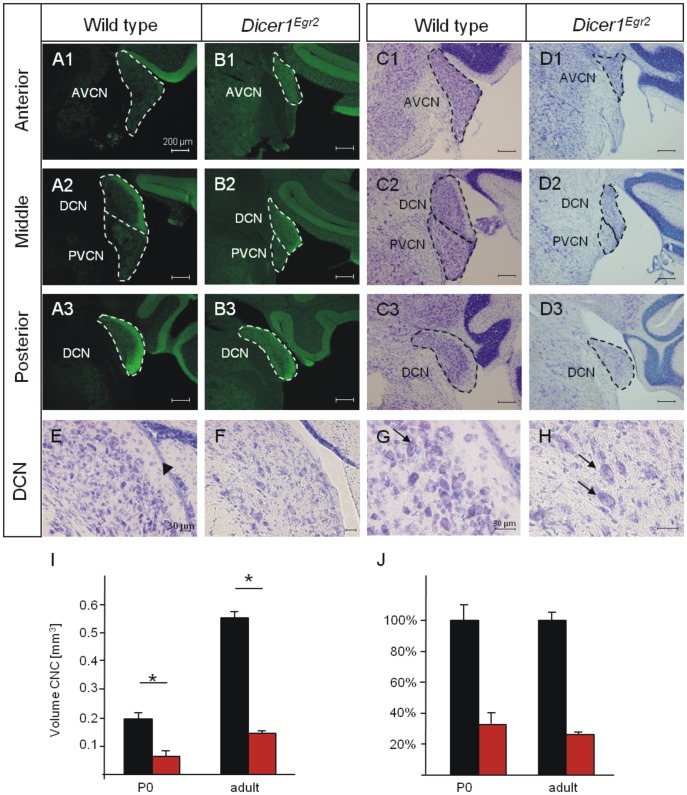
Malformed CNC in adult *Dicer1^Egr2^* mice. ***A,B,*** Vglut1 immunoreactivity in the cochlear nucleus complex of adult wild type (P22– P30) (***A***) or *Dicer1^Egr2^* mice (***B***) at anterior (***A1,B1***), middle (***A2,B2***) or posterior levels (***A3,B3***). ***C,D*** *,* Nissl staining of CNC sections of wild type (***C***) or *Dicer1^Egr2^* mice (***D***) at anterior (***C1,D1***), middle (***C2,D2***) or posterior levels (***C3,D3***) levels. All three subnuclei, i.e. the AVCN, PVCN, and DCN display a reduced size. ***E–H*** Nissl stained sections of the DCN (***E,F***) reveal absence of the microneuronal shell (black arrow), but presence of fusiform cells (black arrows) (***G,H***) in *Dicer1^Egr2^* mice. ***I,*** The volume of the CNC was determined at P0 or adult stage from Nissl stained serial sections. A significant decrease in volume of the CNC was observed in *Dicer1^Egr2^* mice at both ages (2 animals per genotype, aged P0 or P22 and P30) (Mann-Whitney U-test, *, P<0.05). ***J,*** The relative decrease in volume in *Dicer1^Egr2^* mice was similar between P0 and adult mice. AVCN, anteroventral cochlear nucleus; DCN, dorsal cochlear nucleus; PVCN, posterior ventral cochlear nucleus. Dorsal is up, lateral is to the right.

To analyze formation of the CNC in *Dicer1^Egr2^* mice, we performed immunohistochemistry against Vglut1 in mice aged >P22 (adult mice in the following). This presynaptic marker labels the inputs of the spiral ganglion neurons into the CNC [57]. Accordingly, Vglut1 labeling was observed in all three subdivisions, the DCN, the AVCN, and the PVCN, of *Dicer1^fl/fl^* control animals (Fig. 2A). In *Dicer1^Egr2^* mice, all three nuclei were labeled but appeared decreased in size compared to control animals (Fig. 2B). Most evident, the granular cells constituting the microneuronal shell of the CNC [58]–[60] are absent in the CNC (Fig. 2A,B). Nissl stained sections confirmed the loss of these cells in *Dicer1^Egr2^* mice (Fig. 2E,F). This is in agreement with the origin of granular cells in r3 and r5 [16]. To determine whether other cell types with ascertained embryonic origin in these two rhombomeres were absent as well, we analyzed fusiform cells [16]. They represent large output neurons from the DCN and are situated beneath the microneuronal shell. Due to their large size, they can readily be identified in Nissl stained sections. In contrast to granular cells, these large output neurons were present in both wild type and *Dicer1^Egr2^* mice (Fig. 2G,H). These data indicate different dependency of CNC neurons on Dicer or different effectiveness of recombination [44], [45].

**Figure 3:**
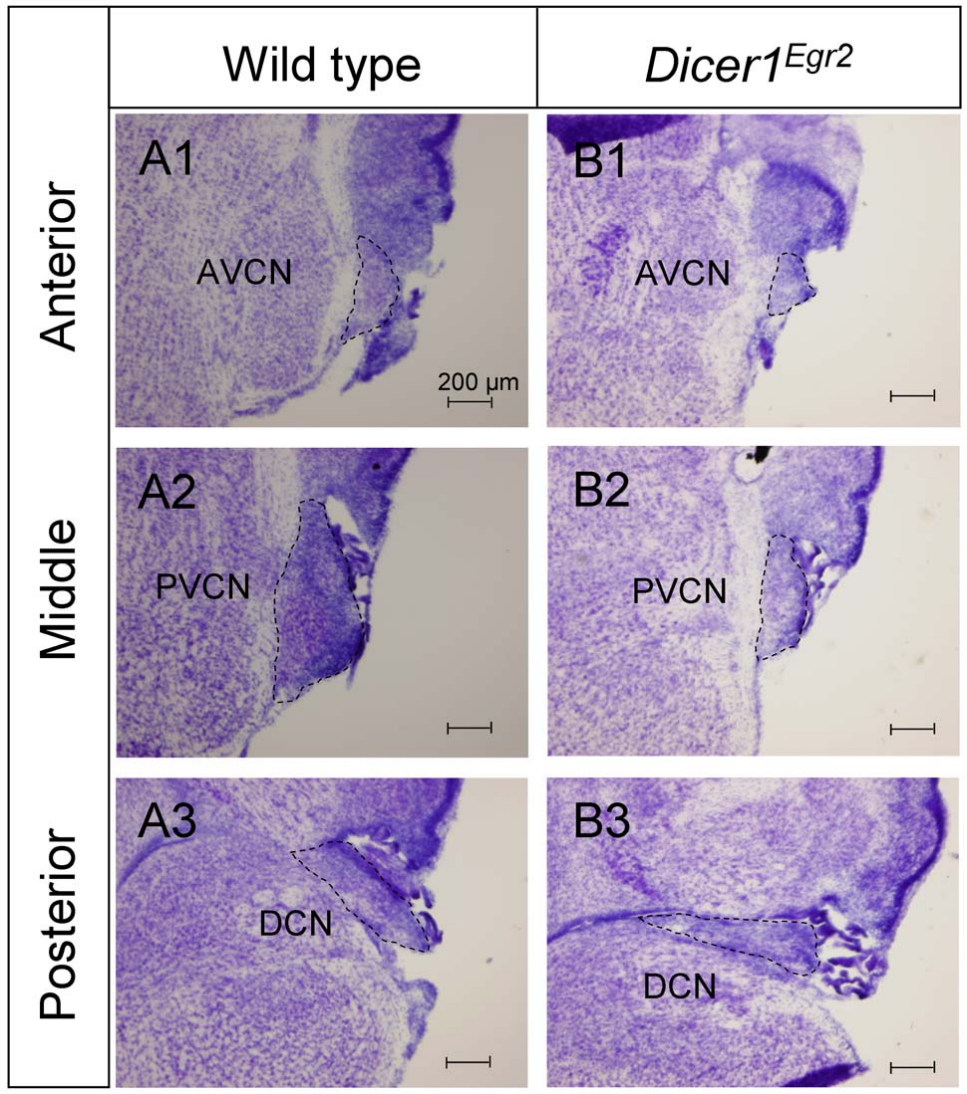
Malformed CNC in P0 *Dicer1^Egr2^* mice. ***A,B,*** Nissl stained sections of the cochlear nucleus complex of wild type (***A***) or *Dicer1^Egr2^* mice aged P0 (***B***) at anterior (***A1,B1***), middle (***A2,B2***) or posterior (***A3,B3***) levels. All three subnuclei, i.e. the AVCN, PVCN, and DCN display a reduced size. AVCN, anteroventral cochlear nucleus; DCN, dorsal cochlear nucleus; PVCN, posterior ventral cochlear nucleus. Dorsal is up, lateral is to the right.

Next, we quantified the volume change in Nissl stained coronal sections (Fig. 2C,D). The lack of the microneuronal shell precluded delineation of the tripartite division in *Dicer1^Egr2^* mice at the transition between the different nuclei. We therefore restricted our volume analysis to the entire CNC. The volume was significantly decreased by 73.5% in *Dicer1^Egr2^* mice compared to control animals (WT: 0.55±0.03 mm^3^; *Dicer1^Egr2^* 0.15±0.01 mm^3^, *P* = 0.021) (Fig. 2I). To investigate, whether the CNC was already disrupted at birth, we performed Nissl staining in P0 tissues (Fig. 3A,B). Quantification of the CNC volume revealed a reduction by 67.2% (WT: 0.197±0.02 mm^3^, *Dicer1^Egr2^*: 0.065±0.017 mm^3^, *P* = 0.034) (Fig. 2I), which was very similar to the decrease observed in adult tissue (Fig. 2J). These data demonstrate that proper formation of the CNC depends on Dicer.

### *Egr2::Cre*-mediated Loss of *Dicer1* Prevents Formation of the SOC

**Figure 4:**
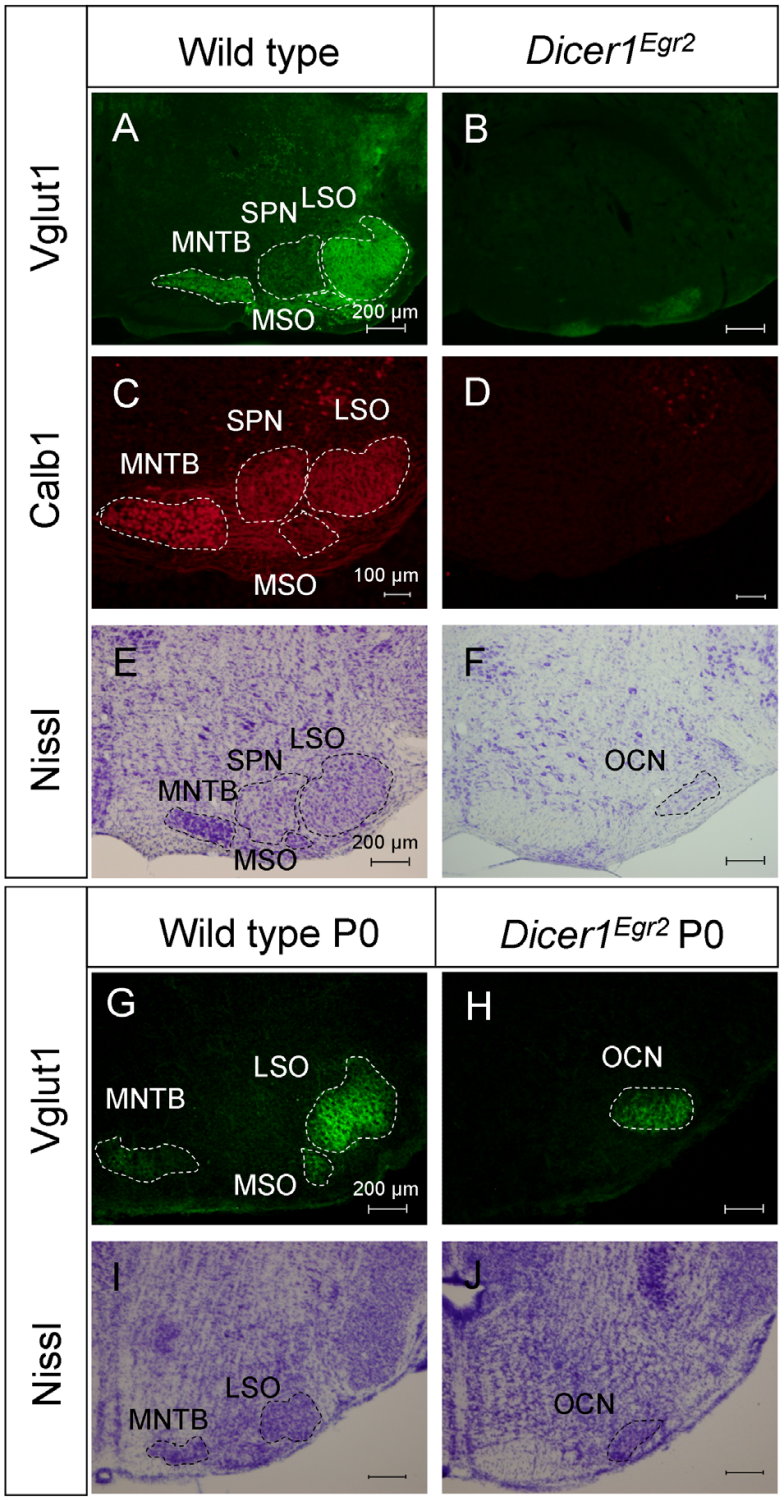
Severe disruption of the SOC in *Dicer1^Egr2^* mice. ***A–F*** Vglut1 immunoreactivity (***A,B***), Calb1 immunoreactivity (***C,D***), or Nissl stained sections (***E,F***) in the superior olivary complex of adult (P22– P30) wild type (***A,C,E***) or *Dicer1^Egr2^* mice (***B,D,F***). Only weak Vglut1 staining is observed in the SOC of *Dicer1^Egr2^* mice and principal nuclei such as the MNTB or the U-shaped LSO cell group are absent in these animals. Calb1 labeling is observed in somata of MNTB neurons and in the neuropil of the LSO, MSO, and SPN in wild type mice. No labeling is observed in *Dicer1^Egr2^* mice. In Nissl stained sections, the cell groups of the MNTB and LSO are recognizable in control mice, but not in *Dicer1^Egr2^* mice. The cell group at the ventral part in *Dicer1^Egr2^* mice corresponds to the olivocochlear neurons. ***G–J*** Vglut1 immunoreactivity (***G,H***), or Nissl stained sections (***I,J***) in the superior olivary complex of P0 wild type (***G,I***) or *Dicer1^Egr2^* mice (***H,J***). Vglut1 staining is restricted to the olivocochlear neurons in the SOC of *Dicer1^Egr2^* mice, and principal nuclei such as the MNTB or the LSO cell group are absent in these animals. In Nissl stained sections, the cell groups of the MNTB and LSO are recognizable in wild type mice, but not in *Dicer1^Egr2^* mice. The cell group at the ventral part in *Dicer1^Egr2^* mice corresponds to the olivocochlear neurons. LSO, lateral superior olive; MNTB, medial nucleus of the trapezoid body; MSO, medial superior olive; OCN, olivocochlear neurons; SPN, superior paraolivary nucleus. Dorsal is up, lateral is to the right.

The severe disruption of the VCN should entail a decreased number of projections into the SOC. We hence performed Vglut1 immunohistochemistry in the SOC. This marker labels the excitatory inputs which originate in the VCN and represent the major projections into the SOC [61]. In adult control animals, the MNTB and the U-shaped LSO were intensely labeled (Fig. 4A). In contrast, only weak immunoreactivity was observed in *Dicer1^Egr2^* animals and the labeling was restricted to very ventral positions in the SOC (Fig. 4B). These data confirm severe reduction of the VCN and its projections and indicate disrupted organization of the SOC.

To further study the SOC, we performed immunohistochemistry against Calbindin 1 (Calb1), a small cytoplasmic calcium binding protein. In agreement with a previous study in the closely related rat [62], Calb1 labeled virtually all somata of MNTB neurons as well as the neuropil of LSO, the MSO, and the superior paraolivary nucleus (Fig. 4C,D). In striking contrast, no Calb1 labeling was observed in *Dicer1^Egr2^* animals in the SOC area. These data indicate absence of major nuclei of the mouse SOC such as the MNTB and LSO. To corroborate this finding, we performed Nissl staining of adult tissue. In agreement with the absence of Calb1 immunolabeling in *Dicer1^Egr2^* animals, we noticed complete lack of the MNTB in coronal sections (Fig. 4E,F). In addition, no U-shaped cell group corresponding to the LSO (Fig. 4E,F) could be detected in *Dicer1^Egr2^* mice.

**Figure 5:**
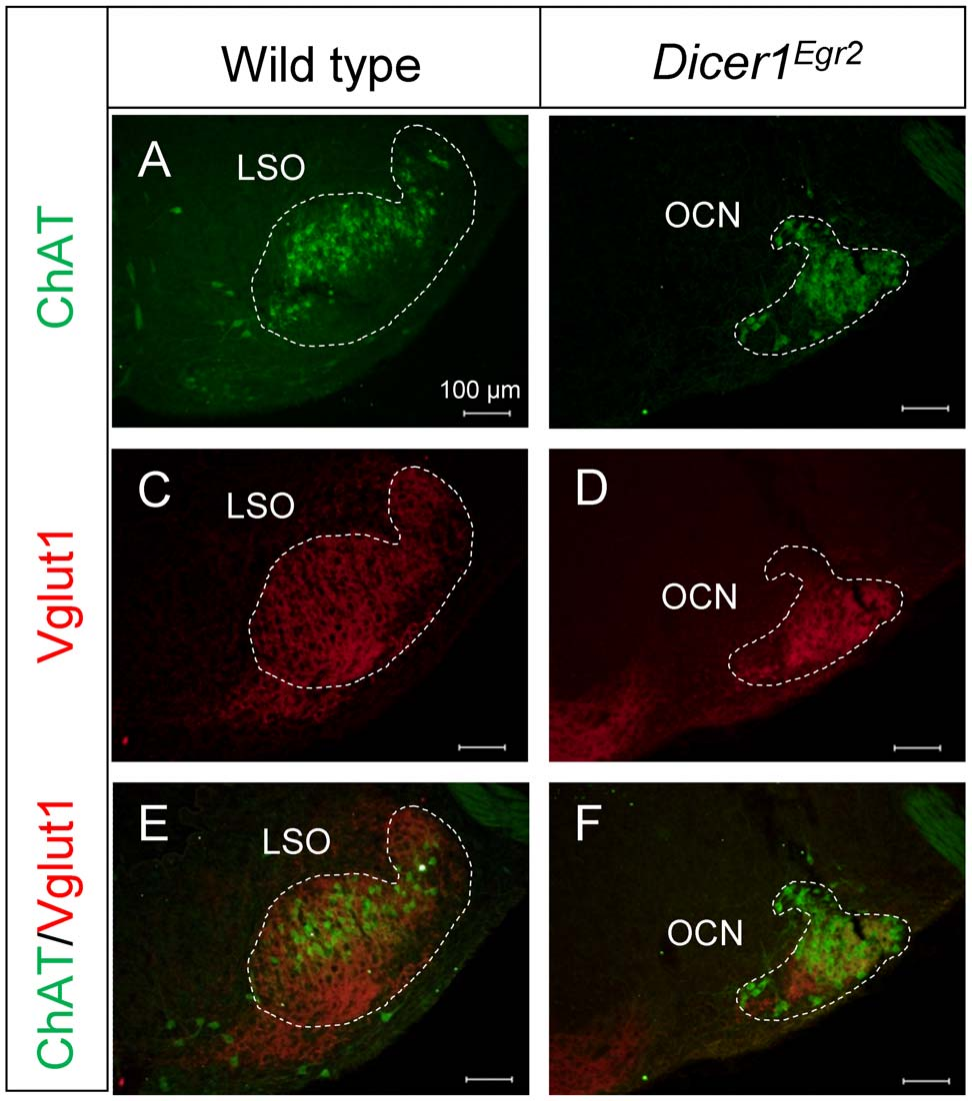
Presence of the olivocochlear neurons in *Dicer1^Egr2^* mice. ***A,B*** ChAT immunolabeling was detected throughout the LSO of wild type mice (***A***), whereas in *Dicer1^Egr2^* mice, ChAT positive cells were restricted to a densely packed ventrolateral cell group (***B***). (***C–F***) The same SOC area was labeled by Vglut1, as revealed in the overlay (***E–F***). Two animals aged P15–P20 were analyzed per genotype. LSO, lateral superior olive; OCN, olivocochlear neurons. Dorsal is up, lateral is to the right.

Vglut1 labeling was detected in two ventral areas of the putative SOC region of *Dicer1^Egr2^* animals (Fig. 4B). The ventromedial area likely consists of the pontine grey, as judged from Nissl stained sections. The ventrolateral cell group was observed in 7–8 Nissl stained slices compared to∼19–21 slices containing the LSO in control animals (Fig. 4F). These cells might represent olivocochlear neurons, which are derived from r4 [63] and hence not targeted in *Egr2::Cre* mice. To determine the identity of this cell group, we performed double labeling experiments using antibodies against Vglut1 and choline acetyltransferase (ChAT), a marker of the cholinergic OC neurons [64], [65]. In agreement with previous analyses [64], [66], we observed in wild type animals ChAT positive neurons intermingled with LSO principal neurons (Fig. 5A). *Dicer1^Egr2^* mice also contained ChAT positive neurons, but labeling was confined to a densely packed ventrolateral cell group (Fig. 5B). The same area was also immunoreactive for the presynaptic marker Vglut1 (Fig. 5C–F). This is consistent with the assumption that OC neurons receive the same input as the principal LSO neurons [2]. These data demonstrate that OC neurons are still present in the SOC. Their organization, however, is altered, likely due to disrupted SOC formation. Together these results reveal a lack of principal nuclei such as the MNTB and the LSO proper in the SOC of adult *Dicer1^Egr2^* mice.

To analyze whether the absence of large parts of the SOC was caused by disrupted histogenesis or was secondary to the malformation of the CNC, we examined P0 *Dicer1^Egr2^* mice as well. In contrast to wild type animals, only weak Vglut1 labeling was present in knockout animals (Fig. 4G,H). Similar to the SOC of adult *Dicer1^Egr2^* mice, immunoreactivity was restricted to the ventral most part of the SOC. To determine the extent of alterations in the perinatal SOC, Nissl stained sections were analyzed (Fig. 4I,J). Alike adult animals, P0 *Dicer1^Egr2^* mice lacked major nuclei such as the MNTB and the LSO. Only a compact cell group was present in the ventrolateral part, which is in agreement with the presence of OC neurons in the SOC from E13.5 on [63]. This cell group was seen in ∼6 slices, compared to ∼15 slices containing the LSO in wild type animals. Taken together, these results identify a crucial role of Dicer for formation of the SOC.

### Secondary Effects of *Egr2-Cre*-mediated Loss of *Dicer1* in the Nuclei of Lateral Lemniscus

**Figure 6:**
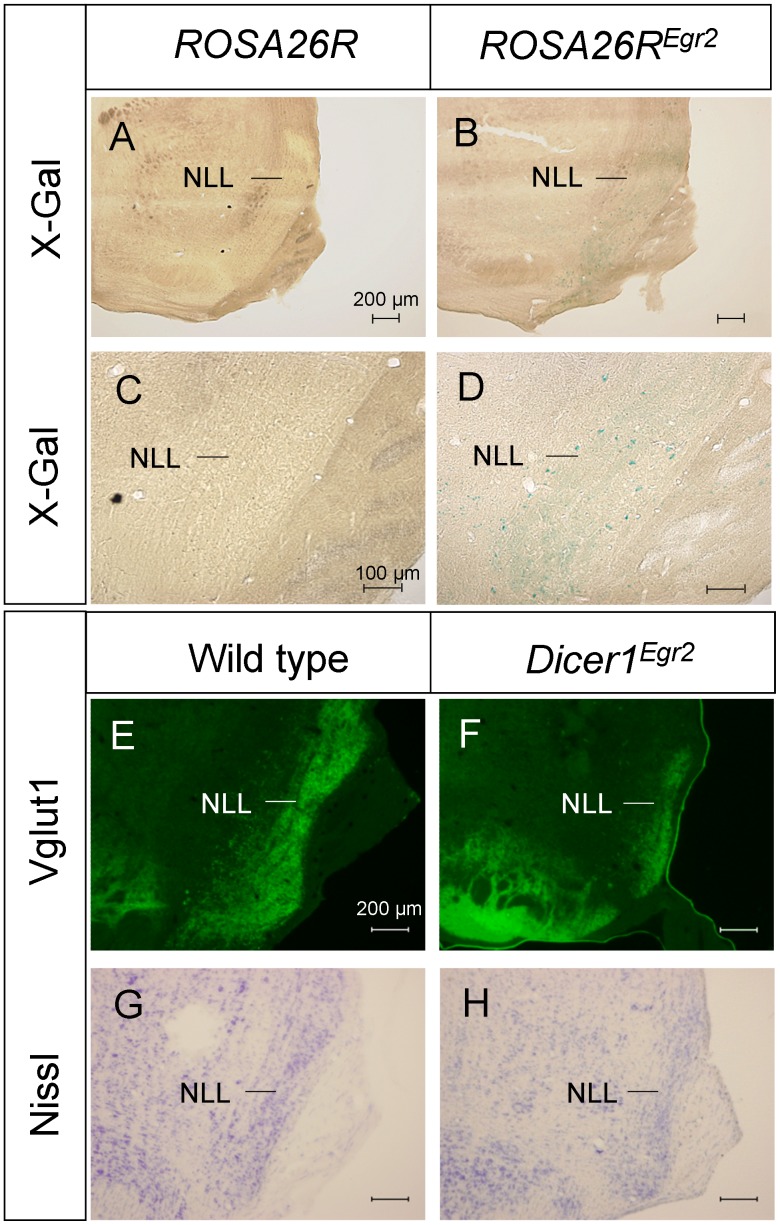
Reduced size of the nuclei of the lateral lemniscus is attributable to secondary effects of *Dicer1* loss. ***A–D***, *Egr2::Cre* mice were crossed with *ROSA26R* mice, resulting in expression of β-galactosidase after Cre-mediated recombination. Only few X-gal positive cells were detected in the nuclei of the lateral lemniscus in the overview (***A,B***) or at high magnification (***C,D***). ***E,F,*** Vglut1 labeling of the nuclei of the lateral lemniscus in adult (P29, 2 animals per genotype) wild type (***E***) or *Dicer1^Egr2^* mice (***F***) revealed reduced size of the nuclei in *Dicer1^Egr2^* mice. ***G,H***, Diminished size of the nuclei in *Dicer1^Egr2^* mice (***H***) was confirmed by Nissl staining (P29, 2 animals per genotype) (***G***). NLL, nuclei of the lateral lemniscus. Dorsal is up, lateral is to the right.

In the ascending auditory pathway, the CNC and SOC project to nuclei of the lateral lemniscus (NLL) [1], [2]. The embryological origin of this auditory center is unknown. Analysis of *Egr2::Cre* mouse paired to *ROSA26R* mice revealed only very few β-galactosidase positive cells in the NLL (Fig. 6A–D). These data reveal that most of the neurons are not descendent from *Egr2* positive cells. To investigate whether disruption of the CNC and SOC causes secondary disruption of this center, Vglut1 immunohistochemistry was performed. Vglut1 stained the NLL in control animals and *Dicer1^Egr2^* mice. In the latter, however, the NLL appeared smaller in size (Fig. 6E,F). To determine whether this was reflecting reduced Vglut1 positive projections from the CNC and SOC or true reduction in size, we additionally performed Nissl staining. This analysis revealed that the NLL was smaller in *Dicer1^Egr2^* mice (Fig. 6G,H). Since this reduction is likely caused by the lack of appropriate innervation from CNC and SOC neurons and not due to a lack of *Dicer1* in the NLL, a detailed quantitative analysis was not performed.

### No Macroscopic Alteration of the CNC in *Dicer1^Atoh7^*

**Figure 7:**
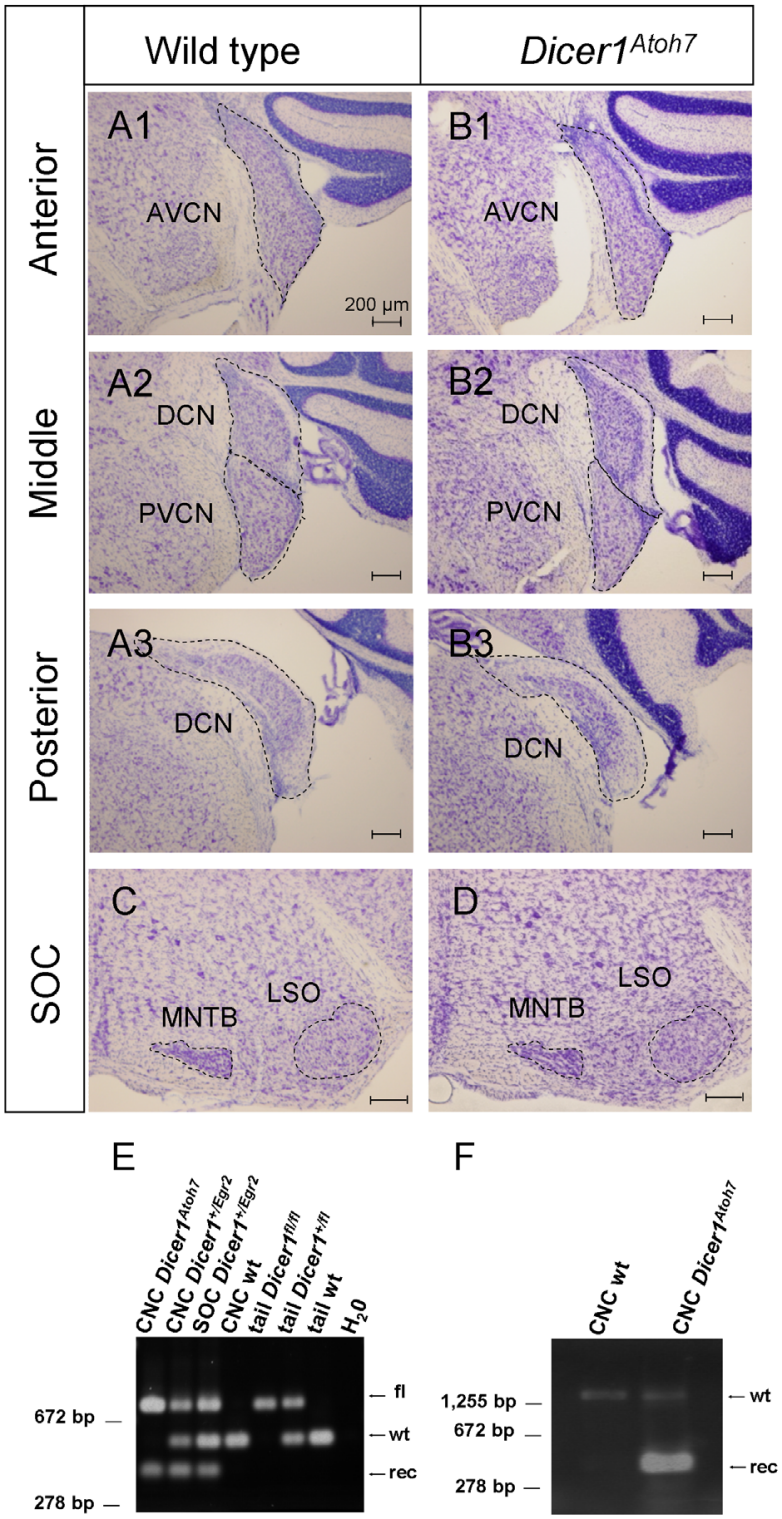
Normal CNC in adult *Dicer1^Atoh7^* mice. *A,B,* Nissl stained CNC sections of adult (P42, 3 animals) wild type (***A***) or *Dicer1^Atoh7^* mice (P42, 3 animals) (***B***) at anterior (***A1,B1***), middle (***A2,B2***) or posterior (***A3,B3***) levels. No difference in shape or size was observed between wild type and *Dicer1^Atoh7^* mice. ***C,D***, Nissl stained sections revealed normal formation of the SOC in *Dicer1^Atoh7^* mice. ***E,*** Genotyping PCR of DNA extracted from the CNC of indicated mouse lines. In homozygous *Dicer1^Atoh7^* mice, two alleles were present: 429 bp (recombined allele) and 767 bp (floxed allele), whereas in the CNC of wild type mice, a 560 bp fragment was amplified corresponding to the wild type allele. ***F,*** RT-PCR analysis of mRNA extracted from the CNC of a wild type or *Dicer1^Atoh7^* mouse. In wild type, only the full length sequence (1,500 bp) was amplified, whereas in *Dicer1^Atoh7^* mice, also the truncated sequence (360 bp) was amplified. Data are representative examples of 2 to 3 biologically independent experiments. These data confirm Cre-mediated recombination in the AVCN. fl, floxed allele; rec, allele after Cre-mediated recombination; wt, wild type allele.

The data obtained so far indicate an essential role of Dicer during histogenesis of the auditory brainstem. *Egr2::Cre* mediates recombination as early as the six-somite stage which is around embryonic day (E) 8 [67], [68]. This early time point precedes the birth date of most auditory brainstem neurons, which is between E9 and E15 [69], [70]. To narrow down the critical period of Dicer action during histogenesis of the auditory brainstem, we wished to ablate *Dicer1* at a later embryonic time point. Since no inducible *Egr2::Cre* mouse line is yet available, we employed an *Atoh7::Cre* mouse line (aka *Math5::Cre*) [52]. On partnering with a reporter mouse line, recombination is observed from E12.5 onwards in bushy cells of the VCN with peak expression at E17 [71]. Since bushy cells represent >90% of all neurons in the VCN [72], we analyzed formation of the VCN in *Atoh7::Cre;Dicer1^fl/fl^* (*Dicer1^Atoh7^* in the following) by Nissl staining of coronal sections in adult animals. In contrast to *Dicer1^Egr2^*, no qualitative evidence for volume reduction was observed in the AVCN or PVCN or the neighboring DCN in *Dicer1^Atoh7^* animals (Fig. 7A,B). In agreement with normal formation of the CNC, the SOC had normal size and organization, as judged from Nissl stained sections (Fig. 7C,D).

*Atoh7* shows low expression in the auditory brainstem compared to the retina [71]. Since recombination requires four Cre molecules acting simultaneously [73], high concentrations of the enzyme are required for *loxP*-mediated recombination [74]. We therefore analyzed whether genetic recombination had occurred in *Dicer1^Atoh7^* animals. Since no antibody is available to specifically detect truncated Dicer protein after recombination, we probed recombination on the genomic and RNA level. Genotyping PCR on DNA isolated from the CNC provided the expected products of 429 bp (recombined allele) and 767 bp (floxed allele) in *Dicer1^Atoh7^* mice, similar to *Dicer1^Egr2^* mice (Fig. 7E). Both products were lacking in the CNC of wild type mice, which displayed a product of 560 bp (Fig. 7E). In addition, RT-PCR on RNA from the CNC of *Dicer1^Atoh7^* mice yielded two products. One cDNA product was of the expected size of 1,500 bp, corresponding to the wild type *Dicer1* allele, and a second product was 360 bp in length, reflecting the deletion of exons 23 and 24 in the recombined allele (Fig. 7F). The shorter cDNA was not observed in control animals (Fig. 7F). These results demonstrate successful recombination in *Dicer1^Atoh7^* mice. The normal structure of the CNC in this mouse line therefore suggests a critical window of Dicer action during embryonic development of the CNC. The precise time window for the action of miRNAs, however, remains to be determined as a delayed disappearance of miRNAs has been reported after loss of Dicer [37], [45], [75].

### Expression of miR-96 in the Brainstem

**Figure 8:**
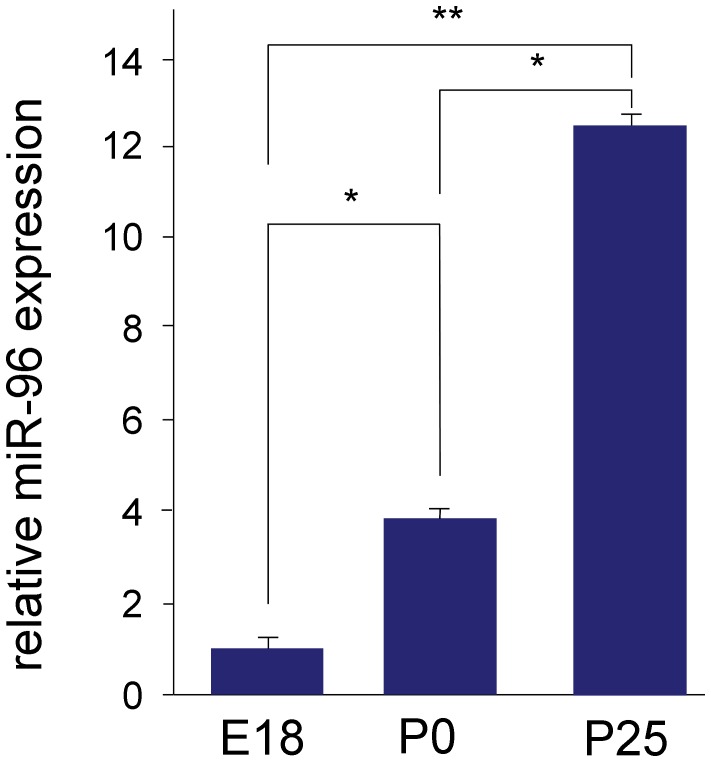
Quantitative RT-PCR analysis of miR-96 in mouse brainstem. qRT-PCR analysis reveals that expression of miR-96 increases in the mouse brainstem with age, when comparing E18, P0 and P25. At least 3 biological repeats were done in triplicates (*t*-test,* P<0.05, ** P<0.01).

In the cochlea, miR-96 is required for proper perinatal development of the cochlea. We recently demonstrated an essential retrocochlear function of the peripheral deafness gene *Cacna1d* for the development of the auditory brainstem [76]. To analyse whether a reotrocochlear role holds also true for miR-96, we determined its expression in the developing brainstem. Quantitative real time-PCR experiments revealed low expression at E18, but up-regulation thereafter. At P0 miR-96 expression increased by 4 fold compared to E18, and at P25 expression increased by 12 fold compared to E18 (Fig. 8). These data suggest a role of miR-96 in postnatal maturation processes of the auditory brainstem but not during histogenesis. Other miRNAs are therefore likely required for proper formation of the auditory brainstem.

## Discussion

### Embryonic Requirement of Dicer for Histogenesis of the Auditory Brainstem

Our analysis of two different mouse lines with differentially timed targeted ablation of Dicer uncovered an essential role of this enzyme for development of the auditory brainstem. We observed striking morphological abnormalities in both the CNC and SOC of *Dicer1^Egr2^* mice. The CNC showed a dramatic volume reduction and the principal nuclei of the SOC such as the MNTB and LSO were absent. These abnormalities were already present at birth, demonstrating disrupted histogenesis of the two auditory brainstem structures. An early requirement of Dicer is supported by our observation, that the VCN developed normal in *Dicer1^Atoh7^* mice. Expression of *Atoh7* in the murine brainstem starts from E12.5 and peaks at E17, whereas *Egr2* is already expressed from E8 onwards. VCN neurons are born in the mouse between E11 and E14 and SOC neurons between E9 and E14 [70]. Thus, *Egr2* expression precedes birth of these neurons by several days, whereas the *Atoh7* expression pattern rather corresponds with the migration of the neurons to their destination in the auditory brainstem [17], [71]. These data indicate a critical time window for Dicer action in the VCN to a period between E8 and E17 (peak expression of *Atoh7* in the VCN). However, recent studies demonstrated also a gap of up to several days between loss of Dicer and miRNA decay [44], [45], [77]. Therefore, the precise time point of miRNA requirement awaits identification of the miRNAs being essential for auditory brainstem development.

A crucial role of Dicer during early embryonic development of central auditory structures is in accord with the lack of the inferior colliculus at E18.5 in mice with *Wnt1::Cre* mediated ablation of *Dicer1* [78]. Furthermore, studies in other neuronal systems such as the neocortex [79], sympathetic neurons [80], forebrain, cerebellum, retina, and ear [37], [44] support a critical role of Dicer during neuronal differentiation [37], [81]. It is therefore likely that the critical time period for SOC neurons, which could not be narrowed down due the lack of suitable Cre-driver lines, is similar to those of other neural populations.

In *Dicer1^Egr2^* mice, the phenotype in the SOC was more dramatic compared to the CNC. Whereas major SOC nuclei such as the MNTB were completely missing, all three major subdivisions of the CNC were present albeit at drastically reduced volumes. Recently, the structural integrity of SOC nuclei was shown to depend on proper formation of the CNC. Disrupted neurogenesis of the AVCN in *Atoh1^Egr2^* mice resulted in increased apoptosis of MNTB neurons, likely due to lack of anterograde trophic support [18]. We consider it unlikely that a similar mechanism contributed to the lack of major SOC nuclei in *Dicer1^Egr2^* mice. In *Atoh1^Egr2^* mice, all SOC nuclei were present at birth and increased apoptosis was noted only postnatally between P0 and P3 [18]. A critical role of innervation in postnatal development is in accord with the observation that neurons of the SOC such as those of the MNTB complete their migration and start to be functionally connected to VCN neurons not earlier than E17 [82], which is only 2 days prior birth (P0 ≈ E19). The absence of major SOC nuclei such as the MNTB or LSO in *Dicer1^Egr2^* mice therefore does not represent secondary loss due to disrupted histogenesis of the CNC. It is rather a direct cause of lack of *Dicer1* in these neurons. Our data thereby are also in excellent agreement with the previous notion, that most SOC neurons are derived from r3 and r5 [18].

In *Dicer1^Egr2^* mice, the only recognizable neurons in the SOC were ChAT positive OC neurons. This is in agreement with their embryonic origin in r4 [63]. These neurons are therefore not Dicer-deficient in *Dicer1^Egr2^* mice. Furthermore, OC neurons arrive several days ahead of the other neurons in the SOC area, as they are present as early as E13 [63]. Survival of OC neurons might therefore be independent of the principal SOC neurons. This observation is in accord with an analysis in *Atoh1^Egr2^* mice, which display a reduced volume of the LSO and MNTB. Similar to the situation in *Dicer1^Egr2^* animals, OC neurons were present, but densely packed at the ventral part of the SOC [18].

### Candidate miRNAs Required for Histogenesis of the Auditory Brainstem

What might be the underling molecular mechanisms of the observed abnormalities in the auditory brainstem? The main function of Dicer is the generation of miRNA (for a notable exception see [83]). Since miR-96 is required for development of the peripheral auditory system, we considered this miRNA as a candidate for the histogenesis of the central auditory system. We therefore studied its expression during development. Due to the small and obscure structure of the perinatal SOC, we had to limit our qRT-PCR to the entire brainstem. This revealed a postnatal up-regulation, suggesting that this miRNA rather plays a role in maturation processes of the auditory brainstem. However, a final conclusion awaits specific expression analysis in the developing SOC and functional studies in *diminuendo* mice, as also low expression of miRNAs might have functional consequences. Nevertheless, other miRNA are likely required for the histogenesis of the auditory brainstem. An attractive candidate might be miR-124, an evolutionary conserved miRNA. Its disappearance in various newborn neurons after deletion of Dicer correlates with cell death (44). Another candidate might be the miR-34 family, which was shown to regulate *Wnt1* [84], and is predicted to target *Atoh1* [85]. Both the *Wnt1* lineage and *Atoh1* lineage of the rhombic lip densely populate the auditory brainstem [16], [18]. Furthermore, Fgf8, an upstream activator of *Atoh1* [86], is regulated by miR-9 [87]. Finally, expression of Hox genes, which are essential for hindbrain organization [88], is also regulated by miRNAs. miR-10 regulates *Hoxb1* and *Hoxb3* [89]. *Hoxb1* descendant cells, which are derived from r4, contribute to the PVCN and DCN, whereas *Hoxb3* is expressed in r5 [88], which largely contributes to the DCN [16] and SOC [18]. Future studies have hence to address the role of these miRNAs in histogenesis of auditory brainstem structures.

In summary, our data demonstrate the crucial embryonic dependence of auditory brainstem formation on Dicer activity. This extends the critical role of this enzyme from the peripheral to the central auditory system. Most likely, the observed phenotype is due to lack of miRNAs governing embryonic development of central auditory structures. Their identification will provide important insights into the genetic program of the auditory brainstem. Equally important might be the role of miRNAs during maturation and function of the auditory system, as indicated by the postnatal up-regulation of miR-96 in the brainstem.
